# Influence of resistant starch resulting from the cooling of rice on postprandial glycemia in type 1 diabetes

**DOI:** 10.1038/s41387-022-00196-1

**Published:** 2022-04-16

**Authors:** Sylwia Strozyk, Anita Rogowicz-Frontczak, Stanislaw Pilacinski, Joanna LeThanh-Blicharz, Anna Koperska, Dorota Zozulinska-Ziolkiewicz

**Affiliations:** 1 Department of Internal Medicine and Diabetology, Poznan University of Medical Sciences, 2 Mickiewicza Str., 60-834 Poznan, Poland; 2 Department of Food Concentrates and Starch Products, Prof. Waclaw Dabrowski Institute of Agriculture and Food Biotechnology—State Research Institute, 40 Starolecka Str, 61-361 Poznan, Poland

**Keywords:** Nutrition, Type 1 diabetes

## Abstract

**Introduction:**

Carbohydrates are one of the macronutrients which have the most substantial influence on glycemic response. The cooling of rice after cooking causes retrogradation of starch, which becomes a non-absorbable product in the human digestive tract.

**Aim of the study:**

This study aimed to assess whether cooling of rice affects postprandial glycemia in subjects with type 1 diabetes.

**Materials and methods:**

The study included 32 patients with type 1 diabetes. Each participant of the study consumed two standardized test meals consisting of long-grain white rice. One of the test meals was served immediately after preparation, and another was cooled for 24 h at 4 °C after preparation and reheated before being served. Postprandial glycemia was measured for 3 h using the FreeStyle Libre flash glucose monitoring system for each patient.

**Results:**

After consumption of the test meal containing rice subjected to the cooling process when compared to fresh rice, a significantly lower value of maximum glycemia (11 vs. 9.9 mmol/L, *p* = 0.0056), maximum glycemic increase (2.7 vs. 3.9 mmol/L, *p* < 0.0001), areas under the glycemic curve (135 vs. 336 mmol/L * 180 min, *p* < 0.0001) and significantly shorter time to peak (35 vs. 45 min, *p* = 0.031) was observed. There was a significantly higher number of hypoglycemic episodes among the patients after consuming test meals with cooled rice compared to fresh ones during 180 min of observation (12(38) vs. 3(9), *p* = 0.0039).

**Conclusions:**

Consumption of rice subjected to the cooling process results in a lower increase of postprandial blood glucose in subjects with type 1 diabetes. At the same time it increases the risk of postprandial hypoglycemia using a standard insulin dose.

## Introduction

Diabetes mellitus is a group of metabolic disorders characterized by hyperglycemia resulting from defects in insulin secretion, insulin action, or both. Almost 10% of all patients with diabetes have type 1 diabetes [1, 2]. The recommended treatment method for type 1 diabetes is intensive insulin therapy. The primary goal in the management of diabetes is to achieve blood glucose values (postprandial and fasting) similar to healthy people to reduce the risk of developing chronic diabetes complications. The most effective method to achieve this goal is a continuous subcutaneous insulin infusion using an insulin pump [3].

Carbohydrates are one of the macronutrients, which have the strongest influence on glycemic response [4]. Rice is one of the most commonly consumed sources of carbohydrates by diabetic patients. Rice is also a daily dietary staple food for more than half of the world’s population [5, 6]. The main carbohydrate in rice is starch. This polysaccharide occurs in a semi-crystalline form in granules and comprises two polymers: amylose and amylopectin [7]. Starch products before consumption are often exposed to heat treatment to increase their availability and digestibility. Starch granules are disrupted by heating in water in a process commonly known as gelatinization, this makes the molecules fully accessible to digestive enzymes [8]. Cooling of cooked starch products decreases the content of available carbohydrates by producing resistant starch. This type of starch is forming in the process called retrogradation. During the cooling of starch, amylose molecules and long branch chains of amylopectin form double helices and lose their water-binding capacity. Double helices of starch molecules are resistant to amylase hydrolysis. The crystallized starch form can resist enzymatic degradation in the small intestine, thus lowering the concentration of digestible starch in cooked starch products [9, 10]. The above phenomenon may benefit patients with diabetes since converting starch into an unavailable form may contribute to lower postprandial glucose values and lower glycemic variability. There are no scientific reports on the effect of retrograded starch on postprandial glycemia in people with type 1 diabetes. There is a need for research evaluating this relationship. Proving the above phenomenon based on the conducted research can bring measurable benefits for metabolic control of diabetes.

This study aimed to assess whether rice cooling affects postprandial glycemia in subjects with type 1 diabetes. Additional goals included evaluation of the effect of cooling rice on the occurrence of hypoglycemic episodes and the degree of feeling hunger, satiety and desire to eat. Moreover, an organoleptic evaluation of rice subjected to the cooling process was performed.

## Materials and methods

### Study group

Thirty-two patients with type 1 diabetes were recruited from the Department of Internal Medicine and Diabetology, the Poznan University of Medical Science, Poland. Inclusion criteria included: 1/type 1 diabetes, age above 18 years, 2/intensive insulin therapy treatment using a personal insulin pump, 3/body mass index below 30 kg/m^2^, 4/HbA1c < 9%, 5/a written consent to participate in the study. Exclusion criteria included: 1/pregnancy, 2/other types of diabetes, 3/eating disorders, 4/food allergies or intolerance to the ingredients of a standardized meal, 5/history of celiac disease, 6/autonomic neuropathy, including gastroparesis, 7/treatment using a personal insulin pump for <3 months.

Body composition analysis was performed using bioelectrical impedance camera BODY COMPOSITION ANALYZER BC-418 MA’s TANITA. Specifically, fat content and free-fat mass was examined.

Moreover, every patient had the following laboratory tests performed: 1/glycated hemoglobin (in whole blood, HbA_1c_), 2/high-density lipoprotein (HDL), 3/low-density lipoprotein (LDL), 4/total cholesterol (TC), 5/triglycerides.

HbA_1c_ was determined by turbidimetric inhibition immunoassay on Cobas 6000 analyzer (Roche Diagnostics, Basel, Switzerland). Other measurements were performed using enzymatic assayson Cobas 6000 analyzer.

All participants were treated with intensive insulin therapy using a personal insulin pump with a bolus calculator function.

Before starting the study, to minimize the influence of the basal rate on blood glucose levels during the tests, each patient tested a basal rate between 1 and 5 pm. In a situation where a nonoptimal basal rate, during this time, caused fluctuations in blood glucose values, the appropriate modification was performed under medical supervision. In the days of the test meal consumption, the basal rate was not changed.

### Study protocol

The Bioethics Committee of Poznan University of Medical Science in Poland approved the clinical study (ethical approval no. 198/18 1.02.2018). The study was designed as a randomized, single-blind crossover study.

Each participant of the study consumed two standardized test meals consisting of long-grain white rice. One test meal was freshly prepared and served immediately after preparation. Another test meal after preparation was cooled for 24 h at 4 °C and then reheated before serving to the patients.

A test meal on a test day could be served only if the patient had no episode of hypoglycemia within 24 h before the study. Before administering the test meal, the participant’s glucose target had to be in the range of 3.9–10 mmol/L. Test meals were always served at the same time, at 2 pm. The time at which the meal was consumed was set at 10 min. The interval between a last meal and a standardized test meal was 5 h. During this time, subjects were only allowed to drink water. Participants were also instructed to avoid unusual vigorous physical activity beginning on the day before each test. Study participants were asked not to exercise before the study until the completion of the test meal.

The patients always consumed the study test meals in the same room and similar circumstances. The patient did not know if the meal consisted of freshly cooked rice or previously chilled. To ensure blindness, the previously cooled test meal was heated to the same temperature as the fresh meal.

10 min before the test meal, patients administered the insulin bolus (Lispro/Aspart) according to carbohydrates exchange factor, correction factor and insulin sensitivity factor. The insulin dose was calculated based on the bolus calculator programmed into the pump. There should be no active insulin left from the previous bolus before serving test meals. All subjects presented stable glycemia, confirmed by the FreeStyle Libre flash glucose monitoring system, before insulin injection and meal consumption.

### Test meals

Long-grain white rice was used for the study. The test meals’ preparation and service procedures were standardized. The test meals were prepared by boiling in water using a Silver Crest induction cooking plate model SIKP 2000 E2.

70 grams of dry product and 280 ml of water were used to prepare the test meal. The rice was dipped into boiling water and cooked for 18 min.

The test meal consisted of 200 g of rice (containing 46 g of carbohydrates) and 100 g of tomato sauce (4 g of carbohydrates).

The tomato sauce did not contain any seasoning. In total, the entire meal contained 50 grams of carbohydrates.

A pre-weighed portion of rice, after preparation, was cooled at 4 °C for 24 h in a refrigerator. After cooling, a portion of rice (200 grams) was reheated by immersion in 250 ml hot water for 3 min.

The rice sample was analyzed for total energy, carbohydrate, protein, fat, ash, total starch, fiber, and water content. Both freshly cooked rice and cooled rice were examined for resistant starch content. Protein content was analyzed using the Dumal method [11]. Fat content was investigated using nuclear magnetic resonance [12]. Starch content was analyzed using the polarimetric method [13]. Ash content was examined using a direct/dry method [14]. Carbohydrate content was determined by difference based on following formula: % Total carbohydrate = [100 − %(Protein + Fat + Moisture + Ash + Fiber)] [15]. Fiber content was analyzed using an enzymatic method [16]. Water content was determined using the oven method [17]. Resistant starch content was analyzed in fresh and cooled test rice (after reheating) using the AOAC 2002.02 method [18]. All analyses, except carbohydrate content, were performed twofold. Arithmetic means of two valuesthat were obtained from the analysis were used as the results.

### Glucose measurements

The postprandial glycemia was assessed over 3 h using the FreeStyle Libre flash glucose monitoring system. Measurements were taken at 5 min intervals. FreeStyle Libre was applied at least 2 days before the first test meal.

### Dietary intake at breakfast before test meals

Patients were instructed to eat the same type of breakfast during all test days to minimize the effect of the first meal on the blood glucose value. Subsequently, the breakfast consumption data before the test meals were collected and analyzed for total energy, carbohydrate, protein, fat and dietary fiber. We used “Dietician 2014” software with the added Polish food database.

### Hypoglycemic episodes

In case of the episode of hypoglycaemia during the test, the experiment was stopped and the patients were asked to consume 15–20 grams of quickly absorbed sugar. Then, the blood glucose measurement was repeated for the next 15 min according to the recommendations of Diabetes Poland. The blood glucose readings below 3.9 mmol/L registered by the FreeStyle Libre sensor or symptomatic hypoglycemia episodes were verified by the measurement of capillary blood glucose using an OptiumXido Abbott Diabetes Care glucose meter. The time of the event occurrence was recorded.

### The organoleptic assessment

During the study, each participant completed an organoleptic evaluation questionnaire that assessed the taste, visual appeal, smell, and consistency of the test meal.

### Hunger feeling, satiety, and desire to eat, assessment

A degree of hunger feeling, satiety and desire to eat was assessed using a proprietary questionnaire based on the Visual Analogue Scale. Participants of the study assessed the intensity of perception of a given trait in numerical values on a scale from 0 to 10. The questionnaire was completed before consuming the test meal and 30, 60, 120, and 180 min after the meal.

### Statistical analysis

Data were analyzed with Statistica PL version 13 (StatSoft, Inc., Tulsa, OK, USA) and MedCalc Statistical Software version 19.3.1 (MedCalc Software Ltd, Ostend, Belgium; https://www.medcalc.org; 2020) for graphical presentation of results. Results are presented as median values (IQR, interquartile range) for continuous variables or numbers and percentages for nominal variables. The normality of continuous variables was tested using Shapiro–Wilk test. Normally distributed variables were compared using paired *t*-test and for continuous variables not meeting its assumptions the Wilcoxon signed-rank test was used.

## Results

### Study group

The mean duration of treatment with an insulin pump was 6.0 ± 3.7 years. The mean HbA1c value was 6.9 ± 0.6%. All participants’ lipid profile parameters were within the normal range (Table 1). The patients did not smoke, had no allergies or food intolerances. There were no chronic complications of diabetes in the study group.

**Table 1:** Subject characteristics (*n* = 32).

Variable	Results (*n* = 32)
Sex F/M *n* (%)	16/16 (50/50)
Age [years]	24 (22.5–29.5)^a^
Duration of diabetes [years]	11.5 ± 5.7
Duration of treatment insulin pump [years]	6.0 ± 3.7
BMI [kg/m^2^]	22.7 ± 2.3
Total fat in body composition [%]	20.1 ± 7.7
HbA1c [%]	6.9 ± 0.6
Total cholesterol [mmol/L]	4.23 ± 0.8
LDL–cholesterol [mmol/L]	1.79 (1.42–2.4)^a^
HDL–cholesterol [mmol/L]	1.93 ± 0.39
Triglycerides [mmol/L]	0.64 (0.53–0.89)^a^

^a^The results were presented as numbers and percentages, mean ± standard deviation or median values (IQR).

### Test meals

The conducted analyzes showed that long-grain white rice contains 435.34 kJ (104 kcal), 2.47 g of protein, 0.1 g of fat, 23 g of carbohydrates, 21.7 g of starch, 0.95 g of fiber, 74.4 g of water and 0.1 gram of ash per 100 g. The resistant starch content in the fresh and chilled test rice was 7.52 ± 0.05 and 11.96 ± 0.04 g/100 g, respectively.

### Glucose response to test meals

**Fig. 1:**
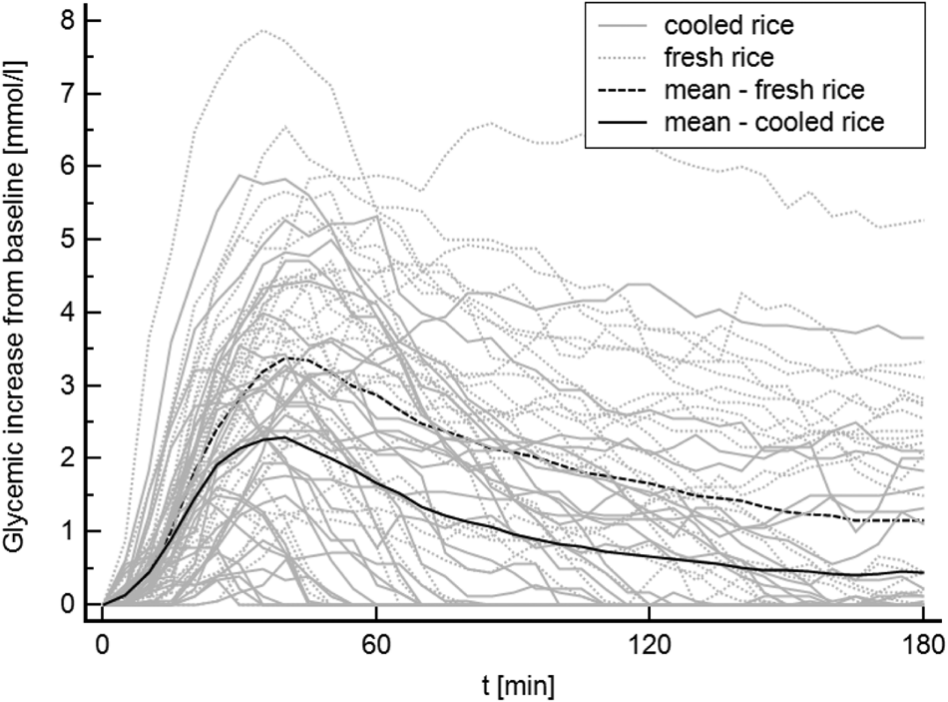
Increase of glycemia after ingestion of meals containing fresh or cooled rice. Values of individual patients (gray lines), mean values for fresh/cooled rice (black lines).

After consumption of the test meal containing the cooled rice compared to fresh one, significantly lower values of maximum glycemia [9.9(9.4–10.9) vs. 11(10.3–11.7) mmol/L, *p* = 0.0056], maximum glycemic increase [2.7(1.5–3.6) vs. 3.9(2.5–4.7) mmol/L, *p* < 0.0001], the incremental area under the glycemic curve [135(34.3–283.9) vs. 336(123.9–486.9) mmol/L * 180 min, *p* < 0.0001] and significantly shorter time to peak of glycemia [35(28–43) vs. 45(35–55) min, *p* = 0.031] were observed (Table 2). An increase of glycemia after ingestion of meals containing fresh or cooled rice is presented at Fig. 1.

**Table 2:** Postprandial glucose responses after test meals.

	Fresh rice	Cooled rice	*p*
Maximum glycemia [mmol/L]	11 (10.3–11.7)	9.9 (9.4–10.9)	**0.0056**
Maximum glycemic increase [mmol/L] 180 min	3.9 (2.5–4.7)	2.7 (1.5–3.6)	**0.0001**
AUC^a^ [mmol/L × 180 min]	335.5 (123.9–486.9)	135 (34.3–283.9)	**0.0001**
TTP^b^ [min]	45 (35–55)	35 (28–43)	**0.031**

The results were presented as median values (IQR) and were compared using Wilcoxon signed-rank test.

^a^AUC—area under the glycemic curve.

^b^TTP—time to peak of glycemia.

Bold values identify statistical significance (*p* < 0.05).

There were no significant differences in the dose of insulin administered to test meals with fresh and cooled rice [5.4 (4.9–6.0) vs. 5.5 (5.0–6.1) units, *p* = 0.92]. There were no significant differences in the basal insulin dose on the days of test meals with fresh and cooled rice [15.8 (13.0–19.9) vs. 15.8 (13.0–19.9) units, (*p* = 0.66].

### Dietary intake at breakfast before test meals

The breakfast, which was the last meal eaten before the test meal for all the patients, did not differ between investigated groups in terms of energy value, protein, fat, carbohydrate, and fiber content. Also the insulin doses administered before breakfast did not differ between both groups (Table 3).

**Table 3:** Dietary intake at breakfast before eating fresh and cooled rice.

	Fresh rice	Cooled rice	*p*
Insulin dose [u]	5.5 (4.0–7.8)	5.5 (4.0–7.8)	0.66
Energy [kcal]	421.9 (313.0–466.5)	410.9 (313.0–466.4)	0.18
Protein [g]	17.9 (13.4–18.7)	17.8 (13.4–18.9)	0.66
Fat [g]	17.3 (11.4–19.6)	17.1 (11.4–19.6)	0.18
Carbohydrate [g]	50.8 (44.1–68.3)	48.1 (42.8–68.3)	0.66
Dietary fiber [g]	7.6 (4.4–8.2)	7.6 (4.5–8.2)	0.66

The results were presented as median values (IQR).

The breakfast, which was the last meal eaten before the test meal for all patients, did not differ between the study groups in terms of energy, protein, fat, carbohydrate and fiber content. Also, pre-breakfast insulin doses did not differ between the two groups

### Hypoglycemic episodes

The number of hypoglycemic episodes was significantly higher among the subjects after consuming a test meal with chilled rice than after consuming fresh rice during the observation period of 180 min (12 vs. 3, *p* = 0.0039) (Table 4).

**Table 4:** Hypoglycemic episodes after test meals with fresh and cooled rice.

	Fresh rice (*n* = 32)	Cooled rice (*n* = 32)	*p*
Hypoglycemic episodes (180 min of observation) [*n* (%)]	3 (9)	12 (38)	**0.0039**
Time of hypoglycemia 180 min of observation) [min]	110 (90–130)^a^	110 (92.5–160)^a^	0.18

The incidence of hypoglycemia was compared using McNemar test.

^a^All values are expressed as numbers and percentages or median values (IQR).

Bold value identify statistical significance (*p* < 0.05).

### The organoleptic assessment of the test meals

No significant differences in the organoleptic assessment between test meals with fresh and cooled rice in terms of palatability [7(6–8) vs. 7(6–8) points, *p* = 0.97], visual appeal of the dish [7(5–8) vs. 7(5–8) points, *p* = 0.72], smell [7(6–9) vs. 7(6–8) points, *p* = 0.32], and consistency [6(5–7) vs. 6(4–7) points, p = 0.53] were noticed. In the opinion of the study subjects, the meals tasted similar.

### Hunger feeling, satiety, and desire to eat assessment after the test meals

No significant differences were observed in the study between test meals with fresh and cooled rice in terms of the sensation of hunger, satiety, and desire to eat 30, 60, 120, and 180 min after the meal.

## Discussion

The original finding of this study was to demonstrate for the first time the beneficial effect of using the specific modification of thermal treatment—cooling of pre-cooked starch products, on postprandial glucose concentrationin subjects with type 1 diabetes. It is related to the phenomenon of resistant starch formation during the cooling of starch products [19, 20]. Yadav et al. showed that multiple heating/cooling cycles of starch products increased the resistant starch content even more [21]. In the present study, the test meal was cooled for 24 h. Only one cooling and reheating cycle was performed for safety and microbiological purity of the analyzed test meals and increasing the suitability for future practical application of the procedure by patients.

To date, the impact of consuming cooled starch products on postprandial glycemia among diabetic patients is unknown. A similar procedure used in the current study was performed by Sonia et al. but carried out on a healthy population [22]. In that study, the glycemic response was analyzed on a freshly cooked white rice (control rice) and cooked white rice that was cooled for 24 h at 4 °C then reheated. The study reported a lower area under the glycemic curve after eating refrigerated rice (125 ± 50 mmol/L * min) than freshly prepared rice (152 ± 48.3 mmol/L * min). Similar results were obtained by Ananda et al. [23]. However, some authors did not confirm the influence of cooled starch products on postprandial glycemia [24, 25].

Our findings are especially valuable,because they were proven on a specific population of patiens with type 1 diabetes, without endogenous insulin secretion.

In our study, a shorter time to peak glycemia was observed with a test meal containing chilled rice compared with fresh rice.

This may help to improve glycemic control as the delayed glycemic peak may better cover with the highest activity of short-acting insulin analogues [26].

Current scientific reports indicate that postprandial glycemia depends not only on the nutritional value of the last meal, but also on the meal consumed earlier. In the study of Meng et al. a lower value of the area under the glycemic curve after the test meal was observed, when a high-protein breakfast preceded it, compared with abreakfast rich in carbohydrates and fat [27]. The study by Granfeldt et al. showed a lower glycemic response when the preceding meal was rich in dietary fiber [28].

In our study, the energy value, protein, fat, carbohydrate and fiber content in the breakfasts preceding the test meals were similar. This minimizes the effect of the previous meal on blood glucose after eating the test meal.

The formation of type 3 resistant starch during the cooling of cooked starch products reduces the content of available and digestible carbohydrates in a meal [19]. In our study, the greater number of hypoglycemic episodes could have been due to the use of the same insulin dose for both test meals. To reduce the risk of hypoglycaemia in patients with insulin-dependent diabetes, consideration should be given to reducing the dose of insulin per meal with chilled rice. It can be assumed that for every 100 grams of chilled rice, the digestible carbohydrate content is reduced by about 5 grams compared to a freshly prepared product.

Lin et al., who investigated the effect of a meal enriched with resistant starch in patients with type 2 diabetes, noted a reduction in postprandial glycemia without an increased risk of hypoglycemia [29]. However, it should be noted that most subjects in this study were treated with oral medications, while a small percentage of patients used prandial insulin.

Our study subjects also assessed the effect of chilling rice on the quality of the consumed meal.

Similar results as in our study were observed by Stewart et al. when comparing 2 food products in terms of organoleptic assessment, one of which was enriched with resistant type 4 starch [30].

However, different results were obtained in the study by Lu et al., where a higher sensory organoleptic evaluation was observed after consumption of chilled praboiled rice compared to praboiled rice and long-grain white rice served immediately after preparation [31]. Sonia et al. in their research, also did not find differences in the assessment of the taste between freshly served rice, and previously chilled to 4 °C and reheated before serving [22]. Therefore, chilling of starch products prior to consumption does not appear to degrade the palatability of the food while having a beneficial effect on glycemia in patients with type 1 diabetes.

It is believed that the content of resistant starch in the diet has a beneficial effect on reducing the degree of hunger and the desire to eat and increasing the feeling of fullness [30]. During cooling, a small amount of resistant starch is formed [32]. In our study the cooling process did not affect the feeling of hunger, satiety and the desire to eat. Several other studies have also reported similar results to our study [26, 31, 33].

## Research limitation and future implications

In the current study, the FreeStyle Libre Glucose Scanning System was used to monitor blood glucose levels after consuming test meals.The patient had a constant view of his glucose readings with this system. Given access, these patients can try to guess what sample of a test meal they were consuming. A possible solution could be the use of patient-blinded continuous glucose monitoring systems that enable retrospective analysis. Several episodes of hypoglycemia were observed with chilled rice within 180 min of postprandial glucose monitoring. The same insulin dose was administered according to the amount of carbohydrate to the test meals, regardless of the cooling process. It is worth repeating the test to determine the appropriate insulin dose reduction per chilled product to reduce the risk of postprandial hypoglycemia.

## Conclusions

Cooling of rice before consumption by patients with type 1 diabetes is essential to achieve better postprandial glycemia. The obtained results indicated the following conclusions:

1. Subjecting rice to a cooling process reduced the increase in postprandial blood glucose in people with type 1 diabetes.
2. Subjecting rice to the cooling process increases the risk of postprandial hypoglycemia in patients who did not change (reduce) insulin dose.
3. Cooling of rice does not affect the organoleptic assessment of a meal.
4. Cooling of rice does not affect the sensation of hunger, satiety, and desire to eat among patients with type 1 diabetes.
